# The Relationship Between Premorbid Weight Status and Eating Disorder Onset in Adolescents: A Longitudinal Study

**DOI:** 10.1002/eat.70042

**Published:** 2026-01-28

**Authors:** Gabriela Tavella, Kris Rogers, Kay Bussey, Phillipa Hay, Nora Trompeter, Deborah Mitchison

**Affiliations:** ^1^ Body Image and Eating Disorder Academic Network, Graduate School of Health University of Technology Sydney Sydney Australia; ^2^ Lifespan Health and Wellbeing Research Centre, School of Psychological Sciences Macquarie University Sydney Australia; ^3^ Translational Health Research Institute Western Sydney University Sydney Australia; ^4^ Mental Health Services South Western Sydney Local Health District Campbelltown New South Wales Australia; ^5^ Population, Policy and Practice Research and Teaching Department, Institute of Child Health University College London London UK

**Keywords:** adolescents, BMI, eating disorders, higher weight status, risk factors

## Abstract

**Objective:**

Adolescents with higher weight status (HWS; body mass index > 85th percentile adjusted for age and sex) are at greater risk of eating disorders (ED). This study examined factors associated with HWS adolescents' increased risk and how weight status interacts with other risk factors to influence ED onset.

**Method:**

Australian adolescents (*N* = 1333; 51.2% female; 11–19 years) completed questionnaires examining ED symptoms and established and emerging risk factors (sex, age, premorbid weight/shape concerns, psychological distress, dieting, and weight‐related bullying) at two timepoints 12 months apart (Waves 1 and 2).

**Results:**

Compared to lower weight peers, those with HWS had greater odds of ED onset when adjusting for sociodemographic variables (OR = 2.96, 95% CI [1.84, 3.93]), other ED predictors (OR = 2.18, 95% CI [1.46, 3.25]), and experiences of weight‐related bullying (OR = 2.20, 95% CI [1.47, 3.28]). The effect of weight status was less pronounced once premorbid dieting, weight/shape concerns, and psychological distress were accounted for, with evidence that weight/shape concerns (OR = 1.17, 95% CI [1.05, 1.30]) and psychological distress (OR = 1.33, 95% CI [1.17, 1.51]) were independently associated with ED onset. No moderation effects were observed.

**Discussion:**

Higher levels of premorbid weight/shape concerns and psychological distress for those with HWS appear to contribute to greater ED risk and should be considered for screening and targeted in prevention.

## Summary

- Higher weight status is a risk factor for eating disorder onset in adolescents.
- We examined how higher weight status interacts with other risk factors in the development of eating disorders in adolescents.
- Results indicated that higher levels of psychological distress and weight/shape concerns might account for why those with higher weight are at greater risk.
- Premorbid weight/shape concerns and psychological distress should therefore be considered when screening for eating disorders in those with higher weight status and targeted in prevention.

## 1 Introduction

Targeting eating disorder (ED) risk factors present during adolescence is critical given that this age represents the peak time for ED onset (Favaro et al. 2019). Risk factors with the strongest replicated evidence for transdiagnostic ED onset include female sex (Suarez‐Albor et al. 2022; Weissman 2019), psychological distress (i.e., an overall negative emotional state characterized by symptoms of depression, anxiety, and stress; Bakalar et al. 2015; Keel and Forney 2013; Stice 2002), higher weight and shape concerns (Bakalar et al. 2015; Glashouwer et al. 2019; Keel and Forney 2013), and unhealthy weight control behaviors, especially strict dieting (Bakalar et al. 2015; Stice 2002). Another risk factor consistently shown to be associated with adolescent ED development is premorbid (i.e., occurring prior to the onset of a disorder) higher weight status (HWS; Bakalar et al. 2015; Suarez‐Albor et al. 2022; Wichstrøm 2000), defined in adolescence as a body mass index (BMI) above the 85th percentile after adjusting for child age and sex, and thus in the “overweight” (≥ 85th percentile) or “obese” (≥ 95th percentile) range (Kuczmarski et al. 2000)—hereto referred to as “higher weight status” (HWS). For example, both retrospective and prospective studies have identified that those with EDs were more likely to report having obesity in adolescence (Hilbert et al. 2014; Wichstrøm 2000), while a recent systematic review of cross‐sectional and longitudinal research found higher weight in adolescents to be a key factor associated with ED symptomology across several of the studies reviewed (Suarez‐Albor et al. 2022). Despite more than a third of adolescents with restrictive EDs having a premorbid history of HWS (Lebow et al. 2015; Sawyer et al. 2016), EDs are under‐researched, under‐diagnosed and under‐treated among those at higher weights (Lebow et al. 2015; Ralph et al. 2022). The aim of this study was therefore to investigate the contribution of HWS and other known ED predictors in risk models for adolescent onset of EDs.

Adolescents with HWS experience other ED risk factors at greater frequency/severity than peers (Goldschmidt et al. 2008). For example, adolescents with HWS exhibit higher levels of weight/shape concerns (Neumark‐Sztainer et al. 2002; Thompson et al. 2006), psychological distress (Kubzansky et al. 2011; Steptoe and Frank 2023), and unhealthy weight control behaviors (Hayes et al. 2018; Lampard et al. 2016; Thompson et al. 2006). This higher level of exposure to the most potent ED risk factors may explain why adolescents with HWS are more likely to develop EDs—though this has not yet been directly tested. Only two longitudinal studies to date have examined ED onset specifically in adolescents with HWS, with both utilizing data from the Project EAT (Eating and Activity in Teens and Young Adults) Cohort in the United States. In the first study (Neumark‐Sztainer et al. 2009), factors present during early/middle adolescence that predicted ED behaviors after 5 years for those with HWS included overvaluation of weight, exposure to weight loss‐related magazine articles, and using unhealthy weight control behaviors. In a follow‐up study of the same cohort (Goldschmidt et al. 2015), increases in body dissatisfaction from Time 1 (early/middle adolescence) to Time 2 (middle adolescence/early young adulthood), and levels of body dissatisfaction and depressive symptoms at Time 2, predicted ED behaviors at Time 3 (early/middle young adulthood). While informative in characterizing ED risk for adolescents with HWS, both studies were restricted to a subsample of HWS adolescents, precluding an analysis of HWS itself as a risk factor and its potential interaction with other risk factors.

Aside from those factors mentioned above, another potential risk factor with particular relevance to those with HWS is weight stigmatization. For adolescents, weight stigma is most often experienced through weight‐related bullying perpetrated by peers (Eddy et al. 2007; Matthews et al. 2022). Weight‐related bullying has been found to prospectively predict increased ED psychopathology in some adolescent studies (see Day et al. 2022), with occurrences of weight‐related bullying more common among adolescents with HWS (Koyanagi et al. 2020; Puhl and Lessard 2020). Experiences of weight stigma may increase ED onset risk through several mechanisms. For instance, the Tripartite Influence Model (Thompson et al. 1999) posits that messages from peers, parents and the media are internalized, which results in increased body dissatisfaction and disordered eating risk (Paterna et al. 2021). Alternatively, the Cyclic Obesity/Weight‐Based Stigma (COBWEBS) Model (Tomiyama 2014) suggests that experiences of weight stigma result in physiological and psychological stress responses, which can lead to maladaptive coping behaviors such as restrictive or emotional eating.

## 2 Aims

This study aimed to examine premorbid HWS as an ED risk factor in an Australian adolescent cohort from the EveryBODY Study (Mitchison et al. 2019; Trompeter et al. 2018). It was hypothesized that:

1. Premorbid HWS would be positively associated with ED onset 12 months later, replicating past research (Bakalar et al. 2015; Mitchison et al. 2023; Suarez‐Albor et al. 2022);
2. The strength of the association between HWS and ED onset 12 months later would be attenuated once accounting for other ED risk factors including premorbid dieting, weight/shape concerns, psychological distress, and premorbid experiences of weight‐related bullying, based on previous research showing their association with HWS and EDs (e.g., Hayes et al. 2018; Lampard et al. 2016; Thompson et al. 2006);
3. Premorbid weight status would moderate the predictive strength of ED risk factors, indicating that the potency of each risk factor is stronger for those with premorbid HWS.

## 3 Method

### 3.1 Participants and Procedures

Data were used from Wave 1 (collected in 2017) and Wave 2 (collected in 2018) of the EveryBODY Study. Detailed descriptions of the study procedures have been published elsewhere (Mitchison et al. 2019; Mitchison et al. 2023; Trompeter et al. 2018). In brief, 13 secondary schools in the Hunter and Sydney regions of New South Wales, Australia, participated in Wave 1, with eight of these schools also participating in Wave 2. Parents provided passive informed consent (i.e., could actively opt their child out of the study), and students provided their assent. Participating students completed a survey comprised of several questionnaires during class time. The EveryBODY study was approved by the Macquarie University Human Research Ethics Committee (HREC) and the New South Wales Department of Education. A waiver of consent was granted by the University of Technology Sydney (UTS) HREC for secondary analyses of the EveryBODY data in the current study (approval no. ETH23‐8665).

**FIGURE 1:**
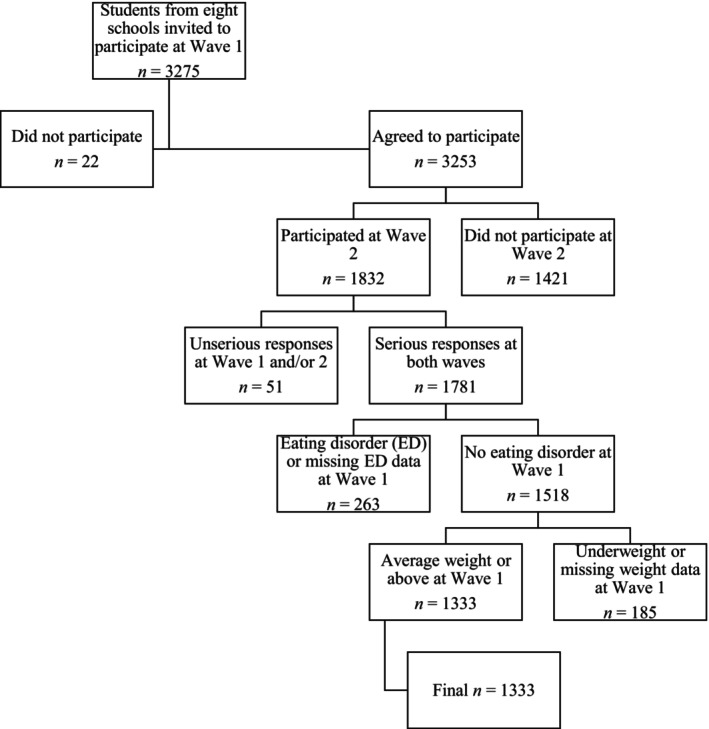
Flowchart of study inclusions and exclusions.

Students invited to participate were adolescents in all grades (year 7 to year 12) aged 11 to 19 years. Inclusion and exclusion criteria are outlined in Figure 1. In summary, from the eight schools who participated in both waves, 3253 students participated at Wave 1, and 1832 of these participated at Wave 2 (56.3% retention rate). There were 374 participants excluded for missing data, unserious responses, or having an ED at Wave 1. Because the study aimed to evaluate the influence of HWS compared to average weight status on ED risk factors, participants who were underweight at Wave 1 (*n* = 125) were also excluded (note: 11 of these participants met criteria for a probable ED at Wave 2). The reason for the exclusion of underweight participants from this study is the likely qualitative differences between the experiences of adolescents who are average weight (i.e., between the 5th and 85th BMI percentile) and underweight (i.e., below the 5th percentile) that influence ED risk (Drosopoulou et al. 2020; Livermore et al. 2020). This left a final sample of *n* = 1333.

### 3.2 Measures

#### 3.2.1 Sociodemographic Questions

The questionnaires collected sociodemographic information including age, school grade, binary sex, gender, country of birth, and postcode. Binary sex rather than gender was used as a predictor in the analyses as one school refused to include questions concerning gender identity in the questionnaire given to their students. Postcodes were later converted into indices representing socioeconomic status using the Australian Bureau of Statistics (ABS) socioeconomic indexes for areas (SEIFA) data (ABS 2018), with lower SEIFA deciles indicative of greater socioeconomic disadvantage.

#### 3.2.2 Weight‐Specific Predictor Variables

##### 3.2.2.1 Premorbid Weight Status

Participants self‐reported current height and weight, from which a BMI was derived (weight [kg]/height [m]) and then converted into a BMI percentile adjusted for age and sex, using the Centers for Disease Control and Prevention guidelines (Centers for Disease Control and Prevention 2017; Kuczmarski et al. 2000). After excluding participants with underweight status (BMI < 5th percentile), the remaining participants were allocated to the higher weight status (HWS; ≥ 85th percentile) or lower weight status (LWS; 5th to 84th percentile) groups.

##### 3.2.2.2 Premorbid Weight‐Related Bullying

As operationalized previously (Day et al. 2021), participants were presented with a standardized definition of bullying (Olweus 1991), and asked how often (0 = not at all to 6 = many times a week) they had been bullied in physical, cyber, relational, and verbal ways in the last school term across 19 items adapted from the Cyberbullying Questionnaire (Gámez‐Guadix et al. 2014). For each item, participants nominated the perceived primary reason for bullying as being due to their “weight/shape” or “other” reasons. Items for which “weight/shape” were selected were summed to create a total weight‐related bullying scale score, with higher scores indicative of more frequent bullying. For the weight‐related bullying scale, McDonald's Omega coefficient (ω) was 0.91 for the HWS group and ω = 0.93 for the LWS group.

#### 3.2.3 Other Predictor Variables

##### 3.2.3.1 Premorbid Dieting

This variable was based on responses to the single author‐derived question “How many days [in the past 4 weeks] have you been on a very strict weight loss diet?” at Wave 1. This item has previously been demonstrated to be a strong predictor in a multivariate model of future ED onset (*β* = 0.16; Mitchison et al. 2023).

##### 3.2.3.2 Premorbid Psychological Distress

This was assessed using scores on the Kessler Psychological Distress Scale (K‐10; Kessler et al. 2002) at Wave 1. The K‐10 characterizes psychological distress as comprising non‐specific negative emotional states, including anxiety, depression, agitation, and fatigue. Scores across the 10 Likert‐type items (1 = none of the time to 5 = all of the time) assess a range of distress symptoms (e.g., “How often did you feel hopeless?”, “How often did you feel that everything was an effort?”, “How often did you feel nervous?”) over the previous month. Consistent with population‐based research (e.g., Kessler et al. 2002; Furukawa et al. 2003; Slade et al. 2011), psychological distress was treated as a continuous variable and scores on each of the 10 items were summed to create a total score (10 to 50), with higher scores indicating greater distress. A cut off of 30 or higher has been suggested as indicative of severe levels of distress (Andrews and Slade 2001). The measure has demonstrated good psychometric properties in Australian adolescent samples (e.g., Bentley et al. 2015) and strong correlations with interview‐based diagnoses of mood and anxiety disorders (Furukawa et al. 2003; Kessler et al. 2002). For this scale, ω = 0.92 for the HWS group and ω = 0.91 for the LWS group at Wave 1.

##### 3.2.3.3 Premorbid Weight/Shape Concerns

This was measured using the combined items of the Weight Concern and Shape Concern subscales of the Eating Disorder Examination Questionnaire (EDE‐Q; Fairburn and Beglin 2008), which assesses the frequency and severity of ED cognitions and behaviors over the preceding 1 month and has been validated for use in Australian adolescents (Mond et al. 2014). Total scores on the combined Weight and Shape Concerns scale represent an average of 12 items examining dissatisfaction, preoccupation with, and overvaluation of weight/shape over the past 4 weeks, with participants reporting the frequency or severity of each experience over the past month on a 7‐point Likert‐type scale (0 = no days/not at all, 6 = every day/markedly), with higher scores indicative of more severe weight/shape concerns. For the combined scale, ω = 0.95 for both the HWS and LWS groups at Wave 1.

#### 3.2.4 Probable Threshold or Subthreshold ED

The presence or absence of a probable DSM‐5 ED diagnosis at Wave 1 and Wave 2 was calculated for each participant using the method employed by Mitchison et al. (2019). EDs included were those associated with body image disturbance and/or behaviors intended to control weight/shape: anorexia nervosa (AN), bulimia nervosa (BN), binge eating disorder (BED), atypical anorexia nervosa (AAN), purging disorder (PD), subthreshold BN, and subthreshold BED. Full descriptions of how each disorder was operationalized are presented in Supporting Information S1.

### 3.3 Statistical Analyses

Missing data analyses were conducted for all variables included in the study, including ED status at Wave 2. Little's missing completely at random (MCAR) test, Mann–Whitney *U* tests (as data were non‐normally distributed), and chi‐square tests were used to examine missing data and explore potential explanations of missingness. Multiple imputation with *m* = 20 was used to replace missing data based on these results (see Supporting Information S2 for details).

Univariate analyses using Mann–Whitney *U* and chi‐square tests were conducted to compare sociodemographic, predictor, and outcome variables between the HWS and LWS groups. A hierarchical logistic regression was then performed with probable ED at Wave 2 as the outcome. In Model 1, weight status at Wave 1 was the sole predictor; in Model 2, sociodemographic variables (sex, age, birth country, SEIFA decile) were entered; in Model 3, other established ED predictors were entered (premorbid dieting, psychological distress, and weight/shape concerns); in Model 4, premorbid weight‐related bullying was added; in Model 5, interaction terms for weight status with each of the other earlier entered ED predictors were added. Akaike information criterion (AIC; Sakamoto et al. 1986) was calculated for each model to compare fit to the data.

Examination of the data prior to analyses highlighted significant floor effects in the distributions of both the premorbid dieting frequency variable and the weight‐related bullying frequency variable, with a large proportion of data points clustered at zero. Both variables were therefore converted into binary categorical variables representing (i) any dieting in the preceding month, and (ii) any weight‐related bullying experienced in the preceding school term, to enhance interpretability and model stability for the remainder of the analyses. While this approach reduces variability, it provides a more robust indicator of exposure when data are highly skewed (Farrington and Loeber 2000; MacCallum et al. 2002). For the regression analyses, birth country was recoded into a binary variable (born in Australia or outside Australia) and SEIFA decile was treated as continuous to simplify regression output and avoid excessive categorization, while K‐10 scores were rescaled by a factor of 5 to ease interpretation of adjusted odds ratios. Analyses were conducted in SPSS Version 28 (IBM Corp 2021), and RStudio (RStudio Team 2021) using the *dplyr* (Wickham et al. 2023) and *readr* (Wickham et al. 2024) packages.

## 4 Results

### 4.1 Missing Data Analysis

Of the data from the final sample (*n* = 1333), 1.49% of total data across Wave 1 and 2 was missing and 189 participants (14.18%) had at least some missing data. All variables included in the analyses had less than 3% missing data, except for the variable assessing weight‐related bullying, which had 11.7% of data missing (due to it being toward the end of the survey). Little's MCAR test was significant (*p <* 0.001), indicating that data was not MCAR. Mann–Whitney *U* and chi‐square tests comparing key sociodemographic and other variables for those with and without missing data indicated that variables had data missing primarily because of participants not finishing the surveys, rather than being due to the nature of the variables themselves. The data were therefore deemed missing at random (MAR) rather than missing not at random (MNAR) (Jakobsen et al. 2017). See Supporting Information S2 for full details. As multiple imputation is appropriate to use on MAR data when less than 40% of data are missing (Jakobsen et al. 2017; Little et al. 2022), multiple imputation (*m* = 20) and analyses of pooled results were therefore used to handle missing data.

### 4.2 Eating Disorder Prevalence and Univariate Comparison of HWS and LWS Groups

Of the 1333 participants, 191 (14.32%) met criteria for a probable ED at Wave 2. BN/subthreshold BN was the most prevalent ED (7.25%), followed by BED/subthreshold BED (4.85%), AN/AAN (3.51%), and PD (2.78%). BMIs at Wave 1 led to 262 participants (19.65% of the sample) being allocated to the HWS group, and 1071 participants (80.35% of the sample) being allocated to the LWS group. A greater proportion of the HWS group (21.68%) met probable ED criteria at Wave 2 compared to the LWS group (12.53%), with the effect size being small (*χ* ^*2*^_(1,1333)_ = 13.69, *p* < 0.001, Cramér's *V* = 0.10).

Results of the univariate comparison of the HWS and LWS groups on Wave 1 variables are displayed in Table 1. Those in the HWS group were more likely to be male, with the effect size being small (Cramér's *V* = 0.14), while there were no differences in age, birth country, or socioeconomic status (i.e., SEIFA decile) between the groups. Those in the HWS group had higher weight/shape concerns and were significantly more likely to have experienced weight‐related bullying in the preceding term. Both effects were small (largest effect size *r* = 0.13). There was no evidence that other variables differed between the groups.

**TABLE 1:** Comparison of sociodemographic and predictor variables reported at Wave 1 between the higher weight status (HWS) and lower weight status (LWS) groups.

Continuous variables^a^
	Medians		Mann–Whitney U	*r*
HWS (*n* = 262)	LWS (*n* = 1071)			
Age	14.4	14.3	137375.00	0.01
Psychological distress	16.0	16.0	122779.00	0.04
Weight/shape concerns	1.0	0.5	113192.50***	0.13

Categorical variables^b^	Percent of group		χ^2^	Cramér's *V*
Binary sex (% female)	37.0	54.7	25.67***	0.14
Ethnicity (% born in Australia)	90.8	84.7	11.89	0.09
SEIFA decile (% in deciles 1 to 5)	65.3	55.0	16.91	0.11
Any dieting in the preceding month (% yes)	10.7	11.7	0.12	0.01
Weight‐related bullying experienced in preceding term (% yes)	18.4	12.6	5.90*	0.07

*Note*: *indicates *p* < 0.05. ***indicates *p* < 0.001.

^a^ As there is no agreed‐upon approach for pooling results from imputed data when using non parametric tests (Eekhout et al. 2017; Won et al. 2009), complete‐case analyses were applied for the univariate analyses of continuous variables (i.e., age, psychological distress, and weight/shape concerns). Percent of missing data for these variables was minimal (i.e., 0.0%, 2.8%, and 0.3%, respectively).

^b^ Degrees of freedom (df) for ethnicity and SEIFA decile chi‐square tests were df = 7 and df = 9, respectively; all other chi‐square tests had df = 1.

### 4.3 The Role of Weight Status in Predicting ED Onset After 1 Year: Univariate, Multivariate and Moderating Effects

Logistic regression results are displayed in Table 2. There was evidence of an association between weight status at Wave 1 and ED onset 1 year later at Wave 2 (Model 1; OR = 1.94, 95% CI [1.37, 2.74]), with greater odds of ED onset for those in the HWS group.

**TABLE 2:** Hierarchical logistic regression output demonstrating relationship between weight status at Wave 1 and probable eating disorder (ED) at Wave 2.^a^

Predictor variable (at Wave 1)^b^	Model 1^c^			Model 2^c^			Model 3^c^			Model 4^c^			Model 5^c^		
	Odds ratio (adjusted)	95% confidence interval		Odds ratio (adjusted)	95% confidence interval		Odds ratio (adjusted)	95% confidence interval		Odds ratio (adjusted)	95% confidence interval		Odds ratio (adjusted)	95% confidence interval	
		Lower bound	Upper bound		Lower bound	Upper bound		Lower bound	Upper bound		Lower bound	Upper bound		Lower bound	Upper bound
Weight status	1.94***	1.37	2.74	2.69***	1.84	3.93	2.18***	1.46	3.25	2.20***	1.47	3.28	2.82	0.95	8.38
Binary sex				4.44***	2.94	6.71	2.83***	1.84	4.36	2.80***	1.82	4.31	3.58***	2.00	6.43
Age				1.19*	1.03	1.36	1.09	0.93	1.26	1.08	0.93	1.26	1.08	0.92	1.25
Birth country				0.92	0.55	1.53	0.93	0.55	1.58	0.93	0.55	1.58	0.91	0.53	1.56
SEIFA decile				1.04	0.96	1.12	1.01	0.93	1.10	1.01	0.93	1.09	1.01	0.93	1.09
Premorbid dieting							1.34	0.84	2.13	1.36	0.86	2.17	1.38	0.80	2.36
Premorbid psychological distress							1.17**	1.05	1.30	1.17**	1.05	1.30	1.17*	1.04	1.32
Premorbid weight/shape concerns							1.33***	1.17	1.51	1.34***	1.18	1.52	1.28**	1.10	1.48
Premorbid weight‐related bullying										0.79	0.45	1.40	0.98	0.52	1.85
Weight status × binary sex													0.52	0.21	1.27
Weight status × dieting													1.02	0.33	3.16
Weight status × psychological distress													0.99	0.78	1.26
Weight status × weight/shape concerns													1.17	0.88	1.54
Weight status × weight‐related bullying													0.49	0.14	1.75

*Note*: *indicates *p* < 0.05. **indicates *p* < 0.01. ***indicates *p* < 0.001.

^a^ Outcome variable was probable threshold/subthreshold ED at Wave 2.

^b^ Categorical predictor variables were weight status (reference category = LWS group), binary sex (reference category = male), migrant status (reference category = Australian born), premorbid dieting (reference category = no dieting) weight‐related bullying (reference category = no bullying).

^c^ Predictor variables added to each model were as follows: Model 1 = Premorbid weight status; Model 2 = Binary sex, age, birth country, SEIFA decile; Model 3 = Premorbid dieting, premorbid psychological distress, premorbid weight/shape concerns; Model 4 = Premorbid weight‐related bullying; Model 5 = Weight status × binary sex, weight status × dieting, weight status × psychological distress, weight status × weight/shape concerns, weight status × weight‐related bullying.

This association remained significant after controlling for sociodemographic variables (OR = 2.96, 95% CI [1.84, 3.93]), with the size of the odds ratio increasing and supporting hypothesis one (Model 2). Binary sex and age were also significantly associated with ED onset in Model 2, with greater odds of ED onset for females (OR = 4.44, 95% CI [2.94, 6.71]) and older adolescents (OR = 1.19, 95% CI [1.03, 1.36]).

In Model 3, when other well‐known ED predictors (i.e., premorbid dieting, psychological distress and weight/shape concerns) were included in the model, the association between HWS and ED onset remained significant (OR = 2.18, 95% CI [1.46, 3.25]). However, a shrinking of the odds ratio compared to the previous model was observed, in line with hypothesis two. Further, alongside HWS, being female (OR = 2.83, 95% CI [1.84, 4.36]), higher levels of psychological distress (OR = 1.17, 95% CI [1.05, 1.30]), and greater weight/shape concerns (OR = 1.33, 95% CI [1.17, 1.51]) were also associated with greater odds of ED onset at Wave 2 in Model 3.

In Model 4, when weight‐related bullying was added as a predictor, little difference was observed in adjusted odds ratios across the other risk factors, and weight‐related bullying was not a significant independent predictor of ED onset. This was contrary to hypothesis two.

Finally, in Model 5, when interactions between HWS and other risk factors were entered into the regression, weight status no longer showed a significant independent association with ED onset. Despite this, none of the interaction effects between weight status and the other risk factors reached significance, which did not support hypothesis three. On the other hand, female sex (OR = 3.58, 95% CI [2.00, 6.43]), higher levels of psychological distress (OR = 1.17, 95% CI [1.04, 1.32]), and greater weight/shape concerns (OR = 1.28, 95% CI [1.10, 1.48]) remained significant independent predictors.

AIC values (see Table 3) were lowest and comparable for Model 3 and Model 4, indicating a better fit to the data when other ED risk factors (but no interactions) were included as predictors in addition to weight status and sociodemographic variables (Burnham and Anderson 2002).

**TABLE 3:** Akaike information criterion (AIC) values for each regression model and AIC difference (ΔAIC) from preceding model.

	AIC^a^	ΔAIC^b^
Model 1	1085.99	
Model 2	1015.90	70.09
Model 3	959.47	56.43
Model 4	960.23	−0.76
Model 5	965.39	−5.16

^a^ AIC values were calculated using average log‐likelihood values across imputations. Lower AIC values indicate better fit to the data.

^b^ ΔAIC absolute values less than 2 indicate that both models show equivalent data fit (Burnham and Anderson 2002).

## 5 Discussion

This longitudinal study aimed to examine the factors associated with greater risk of ED onset for adolescents with HWS, and how HWS interacts with other risk factors to influence ED development. As expected, HWS predicted adolescent ED onset 12 months later when controlling for sociodemographic factors, with ED onset more than twice as likely for the HWS group compared to adolescents with lower weight. HWS remained significantly associated with ED onset when controlling for the other known ED predictors of premorbid dieting, psychological distress, and weight/shape concerns, as well as premorbid experiences of weight‐related bullying. In partial support of the second hypothesis, the size of the HWS effect was smaller once other known ED predictors were accounted for. This indicates that HWS adolescents' exposure to these risk factors may partially account for their greater ED risk, and is in accord with previous research showing that they have increased exposure to these risk factors (e.g., Kubzansky et al. 2011; Neumark‐Sztainer et al. 2002; Steptoe and Frank 2023; Thompson et al. 2006). The third hypothesis that HWS would moderate the effect of other risk factors on ED onset was not supported, as no interactions were significant in the final model.

The smaller weight status odds ratio in Model 3, which included other known ED risk factors, compared to Model 2 suggests that the HWS group's greater exposure to these risk factors partially accounted for their greater risk for ED onset. Higher weight/shape concerns likely contributed to the higher ED risk for this group, as weight/shape concerns were found to be a strong predictor of ED onset across the models, and the HWS group had higher levels of premorbid weight/shape concerns than the LWS group (albeit with a small effect size). Supporting this explanation, weight/shape concerns have been shown to mediate the relationship between HWS and psychological problems in early adolescence (Allen et al. 2006; Burrows and Cooper 2002), and the Project EAT cohort studies identified components of weight/shape concerns (i.e., body dissatisfaction and weight/shape overvaluation) as predictive of ED pathology for adolescents with HWS (Goldschmidt et al. 2015; Neumark‐Sztainer et al. 2009). Premorbid psychological distress was also found to consistently predict ED onset, supporting previous research (Bakalar et al. 2015; Stice 2002). However, unlike previous studies (Kubzansky et al. 2011; Steptoe and Frank 2023), the HWS group did not exhibit greater psychological distress than the LWS group in the univariate analysis. This may be explained by the greater proportion of males in the HWS group, as males tend to report lower levels of psychological distress than females (Bentley et al. 2015; Visani et al. 2011). As sex was controlled for in the regression analyses, it is thus possible that higher levels of psychological distress for those with HWS were also partially responsible for the smaller odds ratio for weight status observed in Model 3. Future research examining the independent mediating effects of weight/shape concerns and psychological distress (rather than entering both factors concurrently into a regression model) will help to determine the relative contributions of these factors in accounting for higher ED risk for those with HWS.

Despite the HWS group being more likely to report having experienced weight‐related bullying than the LWS group, bullying was not found to independently predict ED onset in the multivariate analyses. This does not align with previous studies identifying experiences of weight stigma in general (Vartanian and Porter 2016), and weight‐related bullying specifically (Day et al. 2022), as predicting ED pathology. The null findings may reflect our bullying measure being limited to the preceding school term (which does not capture the chronicity and cumulative effects of weight‐related bullying, which can begin in pre‐ or early adolescence; Juvonen et al. 2016; Reulbach et al. 2013). Furthermore, our bullying data was highly skewed, with only a small proportion (13.7% of the total sample) reporting experiences of weight‐related bullying, which is lower than rates reported in other studies (24% to 64% in recent studies; Puhl et al. 2013; Puhl and Latner 2007; Thompson et al. 2020). Alternatively, research in adults indicates that the association between weight stigma and ED pathology is mediated by both body dissatisfaction and psychological distress (O'Brien et al. 2016; Romano et al. 2021). Thus, controlling for levels of psychological distress and weight/shape concerns (which includes body dissatisfaction) across the regression models may explain the non‐significant effect of weight‐related bullying in this study.

The finding that HWS remained a significant, albeit attenuated, predictor of ED onset even when other risk factors were accounted for indicates that there are other biopsychosocial factors not evaluated here that increase ED risk for those with HWS. For instance, low self‐esteem predicts ED onset for the general adolescent population (Colmsee et al. 2021; Suarez‐Albor et al. 2022), and several studies have shown that adolescents with HWS exhibit lower self‐esteem than peers (Griffiths et al. 2010; Moradi et al. 2021). Weight stigmatization perpetrated by sources other than same‐aged peers, such as teachers and parents, may also influence ED risk (Keery et al. 2005; Quiles Marcos et al. 2013). Social media use, which is positively associated with body image concerns and ED pathology (Dane and Bhatia 2023; Sharma and Vidal 2023), may also contribute to higher ED risk for adolescents with HWS. Emerging evidence indicates that those with HWS engage in more excessive social media use than lower weight peers (Oduro et al. 2023), and that high BMI strengthens the relationship between social media use and the development of ED pathology (Dane and Bhatia 2023). Finally, biological factors that increase one's chance of experiencing HWS and also of developing an ED, such as dysregulation of neural reward pathways (i.e., dopaminergic pathways) and/or hunger regulation (i.e., ghrelin) could also contribute to higher ED risk in those with HWS (see Rancourt and McCullough 2015). Longitudinal studies specifically designed to evaluate these biopsychosocial factors are needed to evaluate whether and how they interact with weight status to influence ED risk.

Against expectations, HWS did not significantly moderate the effect of any of the other key predictors (binary sex, premorbid dieting, psychological distress, weight/shape concerns and weight‐related bullying), and the significant weight status odds ratio identified in Models 1 through 4 became non‐significant once interactions were included in Model 5. The interactions may have absorbed some of the variance previously attributed to the weight status main effect, but with the model having insufficient power to detect true interaction effects. Alternatively, inclusion of the interactions may have led to unnecessary overfitting of the model and masked the weight status main effect. The latter explanation seems likely, as Models 3 and 4 showed better fit to the data than Model 5. Post hoc exploratory analysis supporting this explanation is presented in Supporting Information S3, which demonstrated that when each interaction was examined separately, weight status remained a significant independent predictor of ED onset but did not moderate the effect of any other predictor. This result indicates that while HWS independently predicts ED development and adolescents with HWS are more likely to experience some of the other included risk factors, the predictive strength of the strongest risk factors (i.e., being female, or having weight/shape concerns or psychological distress) is not greater among those with HWS.

### 5.1 Clinical Implications and Future Directions

Appropriate ED diagnosis and treatment are currently impeded for adolescents with premorbid HWS (Kennedy et al. 2017), who can experience delays of over 9 months before receiving appropriate ED treatment compared to lower weight peers (Lebow et al. 2015). Early identification of those with HWS at greatest risk of ED development may help to circumvent this issue, and the current findings suggest that screening for weight/shape concerns and psychological distress may help to identify which adolescents with HWS are at greatest risk before ED onset occurs. Targeting premorbid distress and weight/shape concerns through psychotherapy or other means may prevent later ED onset in these individuals, and should be considered by health professionals managing adolescents with HWS, especially those who present for assistance with weight management. A study in college‐aged women demonstrates the potential for such prevention efforts to reduce ED onset. Namely, Taylor et al. (2006) found that a cognitive‐behavioral intervention significantly reduced weight and shape concerns and decreased risk for ED onset in participants with HWS. The current results also indicate that reducing weight/shape concerns and psychological distress should be prioritized in broader community/school‐based prevention efforts for adolescents of all weight groups, as both were found across the regression models to be consistent predictors of ED onset for the whole sample.

As mentioned, other factors not evaluated in this study likely increase ED risk for those with HWS, and identifying these should be a priority of future research to aid prevention efforts. Clarifying these factors will also help to determine whether current etiological models sufficiently account for ED development in those with HWS, or whether a new risk model is needed for this group. Currently, the cognitive‐behavioral ED model (Fairburn et al. 2003) underpins transdiagnostic ED treatment—enhanced cognitive‐behavioral therapy (CBT‐E; Fairburn 2008). Targeting weight/shape concerns (specifically, weight/shape overvaluation) is a key component of CBT‐E. The current findings provide preliminary evidence as to where HWS may fit into this model, by indicating that higher premorbid weight/shape concerns may partially explain ED risk in those with HWS. Further exploration of the nuances of weight/shape concerns in those with HWS may help to increase the effectiveness of CBT‐E for those with HWS. For instance, exploring whether weight overvaluation specifically is increased in those with HWS, and whether weight/shape concerns are equally responsive to treatment for those with and without HWS, would be of both theoretical and clinical benefit.

## 6 Limitations

Although our missing data analyses indicated no meaningful differences on key study variables between participants included in the study with and without missing data, overall attrition from Wave 1 to Wave 2 (43.7%) may limit the generalizability of our findings. The study was also limited by the use of self‐report observational data to evaluate a causal question. Self‐reported height and weight in adolescents can be reliable for estimating weight status at the group level (Strauss 1999), and are “valuable if the only source of (weight status) data” (Sherry et al. 2007), as was the case in the current secondary dataset. However, self‐reported height and weight data may underestimate HWS prevalence (Sherry et al. 2007), and the results of the current study require replication in studies using objective height and weight measurements. Further, the self‐report method used to determine ED status remains to be validated against structured clinical interview data. The study evaluated transdiagnostic risk factors for a range of EDs, an increasingly favored practice due to shared disorder characteristics and frequent migration of individuals across the differing ED categories (Castellini et al. 2011; Curzio et al. 2018). However, this method may have reduced capacity to detect disorder‐specific effects, as there is some evidence that low BMI is a stronger predictor for AN than high BMI (Stice et al. 2017). Despite this, our results found HWS to be a strong predictor of transdiagnostic ED onset, which aligns with previous research indicating that over a third of adolescents with restrictive EDs (i.e., AN and atypical AN) have a premorbid history of HWS (Lebow et al. 2015; Sawyer et al. 2016). Binary sex rather than gender was also used, which precluded an investigation of gender effects. In addition, only one item was used to evaluate dieting frequency, which may not adequately capture the heterogeneous nature of dieting behaviors (Stice and Presnell 2010), and may explain why dieting was not a significant predictor of ED onset here unlike in previous research (Bakalar et al. 2015; Stice 2002). Furthermore, while defining weight status using BMI percentiles is standard practice and facilitates comparison across studies, BMI‐associated labels (i.e., “overweight” and “obese”) can contribute to weight stigma (Papademetriou et al. 2024), and BMI values do not differentiate adiposity from muscle mass nor account for variance across sociodemographic groups. Finally, while adding multiple variables at once across different models was intended to preserve statistical power by reducing the number of models across the regression, this approach limits the ability to isolate and evaluate the individual contribution of each variable at each step. Future research evaluating the independent contributions of these factors in larger samples (to preserve power) would be beneficial.

## 7 Conclusion

Overall, this study showed HWS to be an important risk factor of adolescent ED onset and identified that higher premorbid weight/shape concerns and levels of psychological distress in individuals with HWS may contribute to their increased ED risk. Identifying additional factors that increase ED risk for adolescents with HWS and designing prevention initiatives that target such factors should be a research priority to reduce ED onset rates in this vulnerable group.

## Author Contributions

**Gabriela Tavella:** conceptualization, formal analysis, investigation, methodology, writing – original draft. **Kris Rogers:** conceptualization, formal analysis, writing – review and editing. **Kay Bussey, Phillipa Hay**, and **Nora Trompeter:** methodology, data curation, project administration, writing – review and editing. **Deborah Mitchison:** conceptualization, investigation, methodology, funding acquisition, project administration, supervision, writing – review and editing.

## Funding

This work was supported by a Macquarie University Research Fellowship.

## Disclosure

Use of AI was not involved in any component of preparation of this manuscript.

## Ethics Statement

The EveryBODY study was approved by the Macquarie University Human Research Ethics Committee (HREC) and the New South Wales Department of Education. A waiver of consent was granted by the University of Technology Sydney (UTS) HREC for secondary analyses of the EveryBODY data in the current study (approval no. ETH23‐8665).

## Conflicts of Interest

P.H. has received sessional fees from the Therapeutic Guidelines publication and the Health Education and Training Institute (HETI, NSW), and royalties/honoraria from Hogrefe and Huber, McGraw Hill Education, Blackwell Scientific Publications, BioMed Central, and PLOS Medicine. She was a consultant to Takeda Pharmaceuticals and is a consultant to Tryptamine Therapeutics.

## Supporting information


**Data S1:** Supporting Information.

## Data Availability

Due to the nature of the research, participants of this study did not give written consent for their data to be shared publicly, so supporting data are not available.
