# Supplementary material for: The Relationship Between Premorbid Weight Status and Eating Disorder Onset in Adolescents: A Longitudinal Study
Source: Int J Eat Disord. 2026 Jan 28;59(5):940–51. doi: 10.1002/eat.70042 (PMC13147143; doi:10.1002/eat.70042)
Supplement: Supplementary file 1 — Data S1: Supporting Information. [file EAT-59-940-s001.docx]

**Supplementary Material 1: Operationalization of eating disorder diagnoses**

Operationalization of DSM-5 eating disorder diagnoses.

| Diagnosis | Criteria |
| --- | --- |
| Anorexia nervosa (AN) | Current body mass index (BMI) percentile <10; AND persistent extreme weight control behavior (fasting/strict dieting/detox, self-induced vomiting, laxative misuse, driven exercise, or misuse of insulin or other drugs) OR fear of weight gain OR felt fat over the past 4 weeks; AND extreme weight/shape concerns over the past 4 weeks |
| Probable bulimia nervosa (BN) | At least four objective binge eating episodes in past 4 weeks; AND persistent extreme weight control behavior in the past 4 weeks (fasting/strict dieting/detox, self-induced vomiting, laxative misuse, driven exercise, or misuse of insulin or other drugs); AND overvaluation of weight and/or shape over the past 4 weeks; AND not meeting criteria for AN |
| Probable binge eating disorder (BED) | At least four objective binge eating episodes in past 4 weeks; AND binge eating associated with three or more features (rapid eating, eating until uncomfortably full, non-hungry eating, eating alone, feeling disgusted/guilty/depressed after eating); AND marked distress regarding the binge eating; AND absence of persistent extreme weight control behavior over the past 4 weeks (fasting/strict dieting/detox, self-induced vomiting, laxative misuse, driven exercise, or misuse of insulin or other drugs); AND not meeting criteria for AN or BN |
| Atypical anorexia nervosa (AAN) | Current BMI percentile ≥10; AND lost weight in the past 4 weeks; AND persistent extreme weight control behavior (fasting/strict dieting/detox, self-induced vomiting, laxative misuse, driven exercise, or misuse of insulin or other drugs) OR fear of weight gain OR felt fat over the past 4 weeks; AND extreme weight/shape concerns over the past 4 weeks; AND not meeting criteria for AN or BN or BED |
| Subthreshold BN | At least two objective binge eating episodes in past 4 weeks; AND at least two episodes of extreme weight control behavior in the past 4 weeks (fasting/strict dieting/detox, self-induced vomiting, laxative misuse, driven exercise, or misuse of insulin or other drugs); AND overvaluation of weight and/or shape over the past 4 weeks; AND not meeting criteria for AN or BN or BED |
| Subthreshold BED | At least two objective binge eating episodes in past 4 weeks; AND binge eating associated with three or more features (rapid eating, eating until uncomfortably full, non-hungry eating, eating alone, feeling disgusted/guilty/depressed after eating); AND marked distress regarding the binge eating; AND absence of persistent extreme weight control behavior over the past 4 weeks (fasting/strict dieting/detox, self-induced vomiting, laxative misuse, driven exercise, or misuse of insulin or other drugs); AND not meeting criteria for AN or BN or BED |
| Purging disorder (PD) | No binge eating in the past 4 weeks; AND at least four episodes of purging in the past 4 weeks (self-induced vomiting, laxative misuse, detox); AND not meeting criteria for AN or BN or BED |

*Note.* This table was reprinted with permission from Mitchison, D., Mond, J., Bussey, K., Griffiths, S., Trompeter, N., Lonergan, A., Pike, K. M., Murray, S. B., & Hay, P. (2019). DSM-5 full syndrome, other specified, and unspecified eating disorders in Australian adolescents: prevalence and clinical significance. *Psychological Medicine*, *50*(6), 981-990. <https://doi.org/10.1017/S0033291719000898>

**Supplementary Material 2: Missing data analysis**

Little’s missing completely at random (MCAR) test was conducted to examine whether missing data were MCAR. When data is not MCAR, multiple imputation can be used to provide unbiased estimates of missing data when (i) more than 5% and less than 40% of data is missing from key variables, and (ii) it is plausible that missingness is based on another variable in the dataset (and is therefore missing at random or MAR), rather than being due to differences in levels of the missing variable itself (i.e., being missing not at random or MNAR; e.g., people who are bullied not wanting to respond to questions about bullying) (Jakobsen et al., 2017; Little et al., 2022).

Mann–Whitney U tests (as data were non-normal) and chi-square tests with effect sizes were used to compare participants with and without missing data on key demographic and other variables at Wave 1 and the likelihood of an ED diagnosis at Wave 2 to explore potential explanations of missingness. As displayed in Table S2, there were no significant differences between those with and without missing data in age, binary sex, country of birth, or SEIFA decile at Wave 1, or likelihood of an ED diagnosis at Wave 2. Those with missing data had slightly higher BMI percentile scores and were significantly less likely to have progressed to the end of the Wave 1 survey than those without missing data. Effect size was trivial for the former and large for the latter. The bullying questions were located at the end of the survey, and inspection of the data and Table 1 results indicated that premature survey abandonment at Wave 1 was responsible for the majority of missing data, indicating that data was likely MAR rather than MNAR. Multiple imputation and analyses of pooled results were therefore used to handle missing data. The number of imputations (*m*) was set conservatively to 20 to prevent biased estimates, as recommended in recent research (Bodner, 2008; Graham et al., 2007; Royston, 2004; Von Hippel, 2009).

| Table S2. Comparison of key demographic and other variables between participants with and without missing data. | | | | |
| --- | --- | --- | --- | --- |
| Continuous Variables | Missing  (*n* = 189) | Not missing  (*n* = 1144) | Mann-Whitney U | *r* |
|  | Medians | |  |  |
| Age at Wave 1 | 14.5 | 14.3 | 103770.00 | 0.02 |
| BMI percentile at Wave 1 | 57.3 | 55.9 | **97389.00*** | 0.06 |
| Progress of Wave 1 survey | 70% | 100% | **18780.00***** | 0.74 |
| Categorical variables | Percent of group | | χ^2^ | Cramer’s *V* |
| Binary sex (% female) | 50.3 | 51.4 | 0.04 | 0.01 |
| Birth country (% born in Australia) | 82.5 | 86.5 | 9.70 | 0.09 |
| SEIFA decile (% in deciles 1 to 5) | 62.6 | 56.1 | 13.79 | 0.01 |
| Probable ED at Wave 2 (% yes) | 13.5 | 13.4 | 0.00 | 0.00 |

*Note:* Significant test statistics are bolded, with *p* < .05 indicated by *, and *p* < .001 indicated by ***.

**References**

Bodner, T. E. (2008). What improves with increased missing data mputations? *Structural Equation Modeling: A Multidisciplinary Journal*, *15*(4), 651–675. https://doi.org/10.1080/10705510802339072

Graham, J. W., Olchowski, A. E., & Gilreath, T. D. (2007). How many imputations are really needed? Some practical clarifications of multiple imputation theory. *Prevention Science*, *8*(3), 206–213. https://doi.org/10.1007/s11121-007-0070-9

Jakobsen, J. C., Gluud, C., Wetterslev, J., & Winkel, P. (2017). When and how should multiple imputation be used for handling missing data in randomised clinical trials – a practical guide with flowcharts. *BMC Medical Research Methodology*, *17*(1), 162. https://doi.org/10.1186/s12874-017-0442-1

Little, R. J., Carpenter, J. R., & Lee, K. J. (2022). A comparison of three popular methods for handling missing data: complete-case analysis, inverse probability weighting, and multiple imputation. *Sociological Methods & Research*, *0*(0), 00491241221113873. <https://doi.org/10.1177/00491241221113873>

Royston, P. (2004). Multiple imputation of missing values. *The Stata Journal*, *4*(3), 227–241. https://doi.org/10.1177/1536867x0400400301

Von Hippel, P. T. (2009). How to impute interactions, squares, and other transformed variables. *Sociological Methodology*, *39*(1), 265–291. https://doi.org/10.1111/j.1467-9531.2009.01215.x

**Supplementary Material 3: Additional exploratory analysis**

| Table S3. Hierarchical logistic regression re-run with interactions examined separately rather than concurrently. | | | | | | | | | | | | | | | | | | | |
| --- | --- | --- | --- | --- | --- | --- | --- | --- | --- | --- | --- | --- | --- | --- | --- | --- | --- | --- | --- |
| Predictor variable (at Wave 1) | Model 1 | | |  | Model 2 | | |  | Model 3 | | |  | Model 4 | | |  |  | | |
|  | (Adjusted) Odds ratio | 95% confidence interval | |  | (Adjusted) Odds ratio | 95% confidence interval | |  | (Adjusted) Odds ratio | 95% confidence interval | |  | (Adjusted) Odds ratio | 95% confidence interval | |  |  |  | |
|  |  | Lower bound | Upper bound |  |  | Lower bound | Upper bound |  |  | Lower bound | Upper bound |  |  | Lower bound | Upper bound |  |  |  |  |
| Weight status | **1.94***** | **1.37** | **2.74** |  | **2.69***** | **1.84** | **3.93** |  | **2.18***** | **1.46** | **3.25** |  | **2.20***** | **1.47** | **3.28** |  |  |  |  |
| Binary sex |  |  |  |  | **4.44***** | **2.94** | **6.71** |  | **2.83***** | **1.84** | **4.36** |  | **2.80***** | **1.82** | **4.31** |  |  |  |  |
| Age |  |  |  |  | **1.19*** | **1.03** | **1.36** |  | 1.09 | 0.93 | 1.26 |  | 1.08 | 0.93 | 1.26 |  |  |  |  |
| Birth country |  |  |  |  | 0.92 | 0.55 | 1.53 |  | 0.93 | 0.55 | 1.58 |  | 0.93 | 0.55 | 1.58 |  |  |  |  |
| SEIFA decile |  |  |  |  | 1.04 | 0.96 | 1.12 |  | 1.01 | 0.93 | 1.10 |  | 1.01 | 0.93 | 1.09 |  |  |  |  |
| Premorbid dieting |  |  |  |  |  |  |  |  | 1.34 | 0.84 | 2.13 |  | 1.36 | 0.86 | 2.17 |  |  |  |  |
| Premorbid psychological distress |  |  |  |  |  |  |  |  | **1.17**** | **1.05** | **1.30** |  | **1.17**** | **1.05** | **1.30** |  |  |  |  |
| Premorbid weight/shape concerns |  |  |  |  |  |  |  |  | **1.33***** | **1.17** | **1.51** |  | **1.34***** | **1.18** | **1.52** |  |  |  |  |
| Premorbid weight-related bullying |  |  |  |  |  |  |  |  |  |  |  |  | 0.79 | 0.45 | 1.40 |  |  |  |  |
|  | Model 5 | | |  | Model 6 | | |  | Model 7 | | |  | Model 8 | | |  | Model 9 | | |
| Predictor variable (at Wave 1) | (Adjusted) Odds ratio | 95% confidence interval | |  | (Adjusted) Odds ratio | 95% confidence interval | |  | (Adjusted) Odds ratio | 95% confidence interval | |  | (Adjusted) Odds ratio | 95% confidence interval | |  | (Adjusted) Odds ratio | 95% confidence interval | |
|  |  | Lower bound | Upper bound |  |  | Lower bound | Upper bound |  |  | Lower bound | Upper bound |  |  | Lower bound | Upper bound |  |  | Lower bound | Upper bound |
| Weight status | **3.03**** | **1.55** | **5.92** |  | **2.19***** | **1.42** | **3.37** |  | 1.98 | 0.74 | 5.32 |  | **1.90*** | **1.02** | **3.53** |  | **2.42***** | **1.55** | **3.77** |
| Binary sex | **3.39***** | **1.92** | **6.01** |  | **2.80***** | **1.82** | **4.32** |  | **2.80***** | **1.82** | **4.32** |  | **2.80***** | **1.82** | **4.31** |  | **2.83***** | **1.84** | **4.37** |
| Age | 1.08 | 0.92 | 1.26 |  | 1.08 | 0.93 | 1.26 |  | 1.08 | 0.93 | 1.26 |  | 1.08 | 0.93 | 1.26 |  | 1.08 | 0.93 | 1.26 |
| Birth country | 0.93 | 0.55 | 1.58 |  | 0.93 | 0.55 | 1.58 |  | 0.93 | 0.55 | 1.58 |  | 0.92 | 0.54 | 1.57 |  | 0.93 | 0.55 | 1.58 |
| SEIFA decile | 1.01 | 0.93 | 1.09 |  | 1.01 | 0.93 | 1.09 |  | 1.01 | 0.93 | 1.09 |  | 1.01 | 0.93 | 1.10 |  | 1.01 | 0.93 | 1.09 |
| Premorbid dieting | 1.34 | 0.84 | 2.14 |  | 1.35 | 0.79 | 2.31 |  | 1.37 | 0.86 | 2.18 |  | 1.39 | 0.87 | 2.23 |  | 1.36 | 0.85 | 2.16 |
| Premorbid psychological distress | **1.17**** | **1.05** | **1.30** |  | **1.17**** | **1.05** | **1.30** |  | **1.16**** | **1.03** | **1.31** |  | **1.17**** | **1.05** | **1.30** |  | **1.17**** | **1.05** | **1.30** |
| Premorbid weight/shape concerns | **1.34***** | **1.18** | **1.52** |  | **1.34***** | **1.18** | **1.52** |  | **1.34***** | **1.18** | **1.52** |  | **1.31***** | **1.13** | **1.51** |  | **1.34***** | **1.18** | **1.52** |
| Premorbid weight-related bullying | 0.80 | 0.45 | 1.41 |  | 0.79 | 0.45 | 1.40 |  | 0.79 | 0.45 | 1.40 |  | 0.79 | 0.44 | 1.40 |  | 0.95 | 0.50 | 1.78 |
| Weight status X binary sex | 0.59 | 0.26 | 1.37 |  | - | - | - |  | - | - | - |  | - | - | - |  | - | - | - |
| Weight status X dieting | - | - | - |  | 1.03 | 0.34 | 3.15 |  | - | - | - |  | - | - | - |  | - | - | - |
| Weight status X psychological distress | - | - | - |  | - | - | - |  | 1.02 | 0.82 | 1.27 |  | - | - | - |  | - | - | - |
| Weight status X weight/shape concerns | - | - | - |  | - | - | - |  | - | - | - |  | 1.07 | 0.85 | 1.36 |  | - | - | - |
| Weight status X weight-related bullying | - | - | - |  | - | - | - |  | - | - | - |  | - | - | - |  | 0.54 | 0.16 | 1.79 |
| Outcome variable was probable threshold/subthreshold ED at Wave 2. Categorical predictor variables were weight status (reference category = LWS group), binary sex (reference category = male), migrant status (reference category = Australian born), premorbid dieting (reference category = no dieting) weight-related bullying (reference category = no bullying). Significant odd ratios are bolded, with *p* < .05 indicated by *, *p* < .01 indicated by **, and *p* < .001 indicated by ***. | | | | | | | | | | | | | | | | | | | |
